# A Portable Laser Photoacoustic Methane Sensor Based on FPGA

**DOI:** 10.3390/s16091551

**Published:** 2016-09-21

**Authors:** Jianwei Wang, Huili Wang, Xianyong Liu

**Affiliations:** 1 School of Information Engineering, Robot Technology Used for Special Environment Key Laboratory of Sichuan Province, Southwest University of Science and Technology, Mianyang 621010, China; Liuxianyong@swust.edu.cn; 2 Joint Laboratory for Extreme Conditions Matter Properties, Southwest University of Science and Technology and Research Center of Laser Fusion CAEP, Mianyang 621010, China; wanghl760@163.com

**Keywords:** FPGA, photoacoustic spectroscopy, diode laser, methane

## Abstract

A portable laser photoacoustic sensor for methane (CH_4_) detection based on a field-programmable gate array (FPGA) is reported. A tunable distributed feedback (DFB) diode laser in the 1654 nm wavelength range is used as an excitation source. The photoacoustic signal processing was implemented by a FPGA device. A small resonant photoacoustic cell is designed. The minimum detection limit (1σ) of 10 ppm for methane is demonstrated.

## 1. Introduction

Methane (CH_4_) detection plays an important role in various applications, such as natural gas exploration, coal mining, medical diagnostics and environment monitoring [1,2,3,4,5]. There exist numerous CH_4_ sensing devices based on different detection principles like catalytic combustion, gas chromatography (GC), electrochemical sensors, semiconductor, infrared spectroscopy, etc. [6,7,8]. Compared with the above sensors, the main advantages of laser photoacoustic (PA) gas sensors are the high sensitivity, large dynamic range, without the need for sample preparation and in situ measurement option.

PA detection of trace gases is one of the most sensitive techniques of infrared absorption spectroscopy [9,10,11,12]. The success of PA gas detection crucially depends on the availability and performance of excitation light sources in combination with appropriate detection schemes. There are several factors affecting PA gas detection technology. The two main factors are the light source and the weak signal detection technique. Tunable near-infrared diode lasers and tunable mid-infrared quantum cascade lasers (QCLs) are generally employed as excitation light sources today [13,14,15]. The tunable distributed feedback (DFB) diode laser is the appropriate laser source for portable gas sensors due to its compact shape and stability characteristics [16,17,18,19,20]. The weak signal detection technique for the PA gas detection is another key factor. The use of a commercial lock-in amplifier makes the PA gas detection system achieve high sensitivity. These gas detection systems based on a commercial lock-in amplifier (SR830, Stanford Research Systems, Sunnyvale, CA, USA) are hardly integrated and miniaturized for their relatively large volume. With rapid development of the field-programmable gate arrays (FPGA) technology, the FPGA has extended the application of digital signal processing to a larger field of instrumentation in recent years [21,22,23,24]. The FPGA technology makes the sensors portable, reconfigurable and reprogrammable with the advantages of good low-cost, high integration and high-performance signal processing [25,26,27,28]. The digital lock-in amplifier based FPGA may provide new opportunities for developing portable PA gas detection devices.

In this paper, a portable PA methane sensor based on FPGA in combination with a tunable distributed feedback (DFB) diode laser is developed. The performance of the sensor is demonstrated by the measurements of CH_4_. A detection limit of 10 ppm is obtained.

## 2. Sensor Design

**Figure 1:**
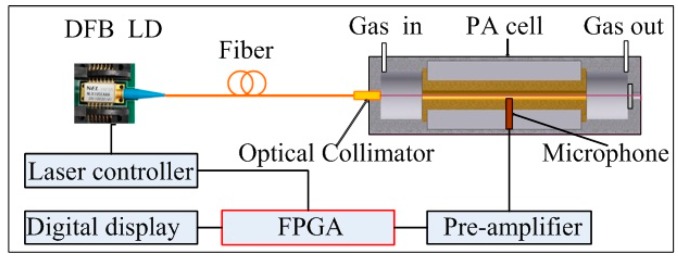
Schematic configuration of the PA methane sensor.

**Figure 2:**
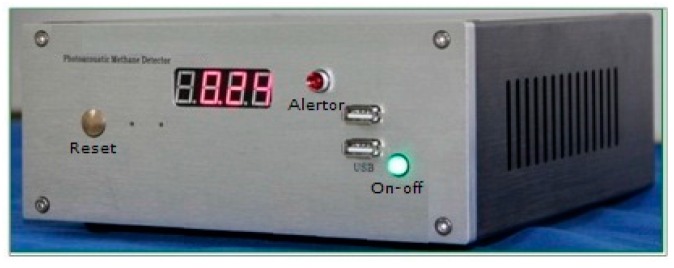
The photograph of this PA methane sensor.

A schematic of the portable PA methane sensor is shown in Figure 1. A tunable DFB, fiber-coupled diode laser (NLK1U5EAAA, central wavelength 1653.72 ± 0.05 nm, 10 mW, FC/PC pigtailed, NEL, Kanagawa-ken, Japan) is used as the excitation light source. The laser beam is collimated using a fiber optical collimator (operating wavelength 1650 nm, Primanex, Shenzhen, China) for direct installation in the resonant PA cell. The DFB diode laser is operated in wavelength modulation mode by the laser controller. Modulation of the laser current is performed by applying a sinusoidal dither to the direct current ramp of the DFB diode laser at half of the PA cell’s resonance frequency. The PA signals are detected by a 22 mV/Pa microphone (EK3033, Knowles Electronics, Chicago, IL, USA) which is placed in the middle of the PA cell resonator. The amplified PA signals by a high input impedance preamplifier (AD8221, Analog Devices, Norwood, MA, USA) are measured using the lock-in amplifier based on FPGA with a time constant 1 s. Gas concentration is displayed on a digital display after data acquisition and processing. A photograph of the portable PA sensor is shown in Figure 2 (220 × 200 × 80).

**Figure 3:**
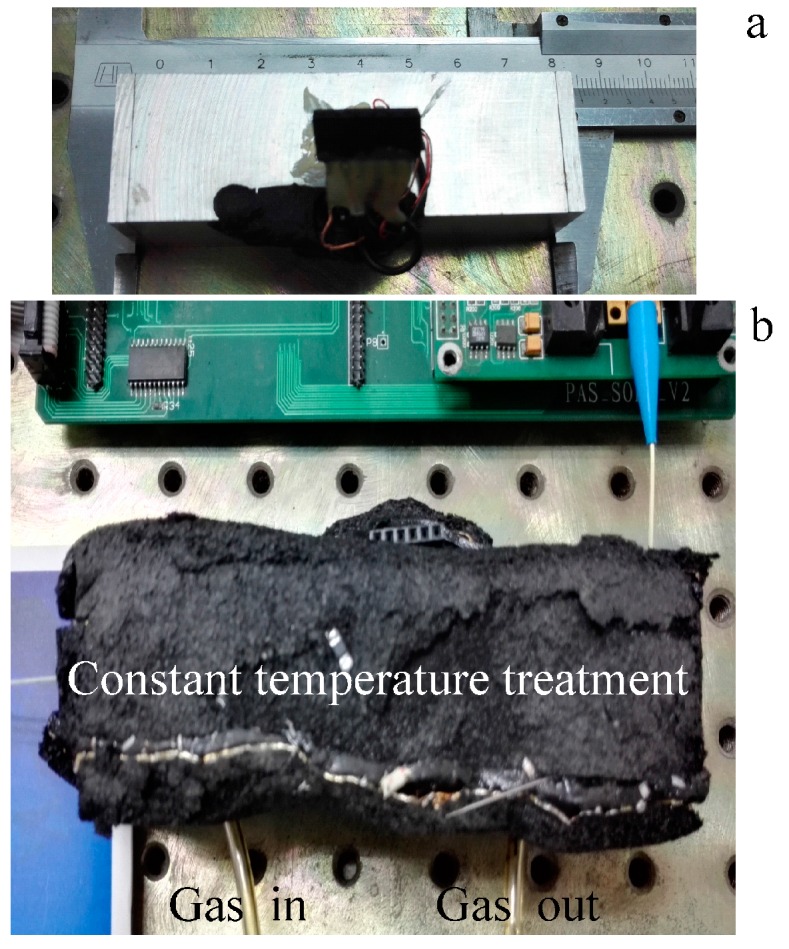
(**a**) The PA cell and (**b**) the constant temperature treatment device.

**Figure 4:**
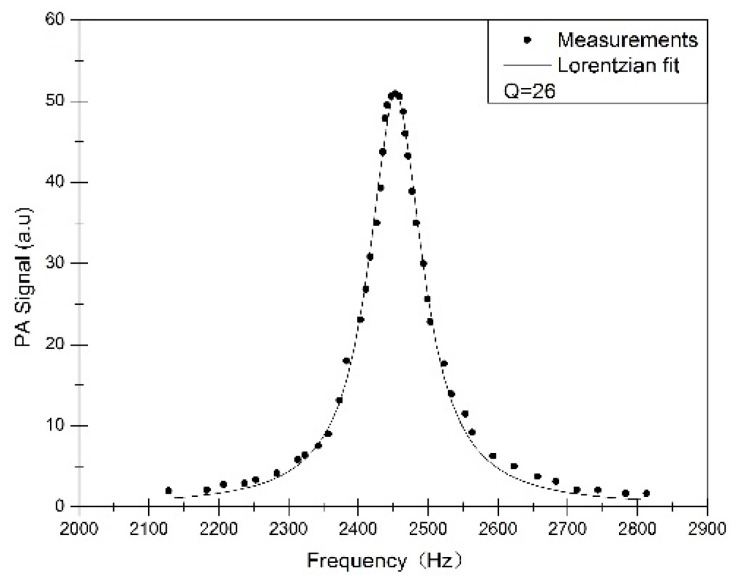
The resonance profiles showing the first longitude resonance frequency is 2450 Hz.

In order to achieve a portable gas sensor, a small resonant PA cell is designed. The shape and size of this PA cell are shown in Figure 3a. The diameter and length of the brass resonator are respectively 4 mm and 36 mm. The length of two buffer volumes is 18 mm, and their diameters are 20 mm. The length of both ends flanges is 2 mm. The end of the PA cell is sealed by a quartz window. The PA cell is highly insulated with special material to reduce the environmental temperature and humidity interferences (see Figure 3b). A temperature controller accurately controls the temperature of PA cell to maintain a temperature of 35 °C. Under this condition the PA cell’s resonance is obtained (see Figure 4). The frequency, half-width and Q-factor of the resonance have been determined by extracting parameters from a Lorentzian fit. For this cell, the resonant frequency is 2450 Hz and the Q-factor is about 26 at atmospheric pressure.

**Figure 5:**
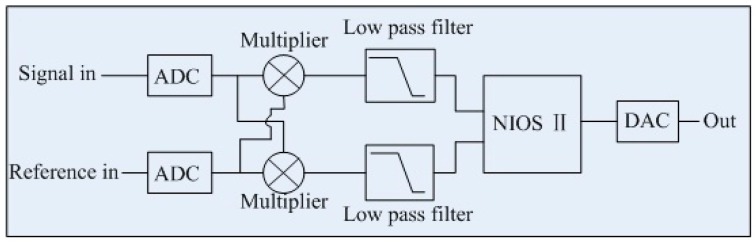
Schematic diagram of the FPGA based lock in amplifier.

The lock-in amplifier based on FPGA can be seen in Figure 5. It is implemented in a ProASIC3 series low cost, low power FPGA (ProASIC3EL, ACTEL, New York, NY, USA). The FPGA is configured on a NIOS II processor (It is the second generation embedded processor architecture for FPGAs.) with C programming language. Implementing components in FPGA allows all operations after analog-to-digital (AD) sampling to be done digitally at high speed. The workflow and each functional modules of the FPGA are coordinated by this NIOS II processor [29,30]. Gas concentration is achieved to implement a computation repeatedly, composed of a series of ordered operations on a set of PA signal data. Concentrations can be directly routed to a digital-to-analog converter (DAC) as well as displayed.

It is assumed that the PA signal is *X1* = *V_s_*cos(*ωt* + *θ*), one reference signal is *R1* = *V_r_*cos(*ωt* + *ϕ*), another orthogonal reference signal is *R2* = *V_r_*sin(*ωt* + *ϕ*). When the PA signal *X1* is separately multiplied by the reference signals *R1* and *R2*, the results are low pass filtered yielding:

(1) $$I=kV_{s}V_{r}\cos\;\theta$$

(2) $$Q=kV_{s}V_{r}\sin\;\theta$$

According to Equations (1) and (2), the phase difference and the amplitude can now be calculated by this NIOS II processor:

(3) $$V_{s}=\frac{1}{kV_{r}}\sqrt{I^{2}+Q^{2}},\;\theta =\text{arctan}(\frac{Q}{I})$$

where *V_S_* is the amplitude the PA signal, *θ* is the phase difference of the PA signal.

The gas sample preparation unit consists of two mass flow controllers (MFC) and two bottles of certified gas (5000 ppm CH_4_ and air). The two MFCs were used to dilute the certified CH_4_ gas. From 100 ppm to 5000 ppm, different concentrations of CH_4_ were generated by the sample gas preparation unit. For reducing excessive flow noise resulting from turbulence in the PA cell, a total flow of 100 standard cubic centimetres per minute (sccm) was used. 

## 3. Results and Discussion

**Figure 6:**
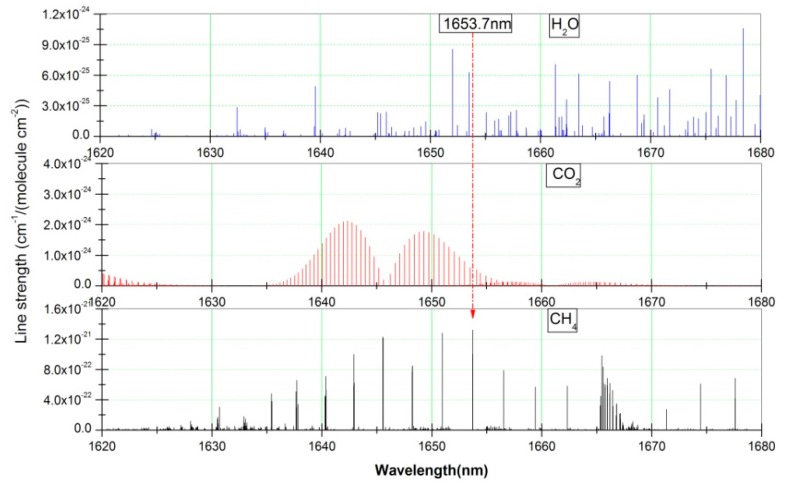
Absorption spectrum of the gases (H_2_O, CO_2_ and CH_4_) obtained from the HITRAN database.

Carbon dioxide (CO_2_) and water vapour (H_2_O) are the most ubiquitous gases in the environment, which have characteristic infrared absorptions. Therefore the spectral overlap interference of none-measured gases in CH_4_ detection needs to be solved. An absorption line at 1653.7 nm is selected for CH_4_ detection by analyzing spectral range of center wavelength and light source characterictics (see Figure 6). The absorption spectrum of H_2_O, CH_4_ and CO_2_ between 1620–1680 nm was obtained from the HITRAN database [31]. Figure 6 shows there is no spectral overlap interference at 1653.7 nm.

**Figure 7:**
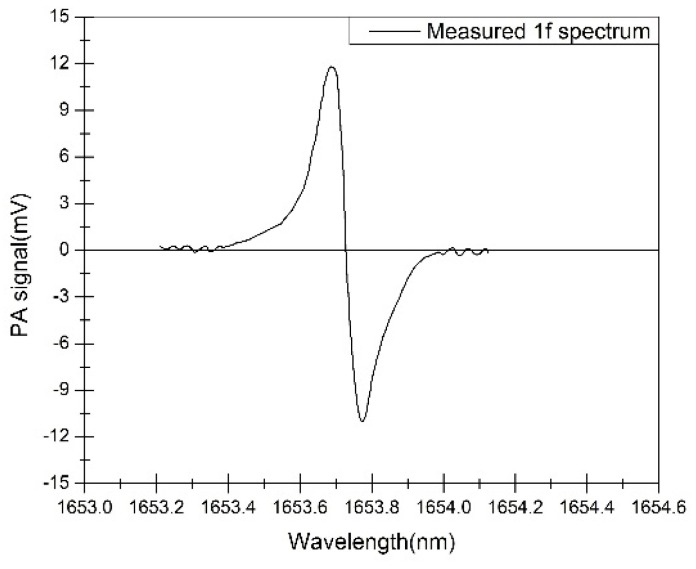
Measured 1*f* spectra of 200 ppm CH_4_.

The DFB diode laser was operated under control of the operating system. The spectrum at 1653.7 nm was obtained by varying the laser operating temperature from 10 to 40 °C. The sensor was used to measure the CH_4_.The measured 1*f* spectra of 200 ppm CH4 is shown in Figure 7.

**Figure 8:**
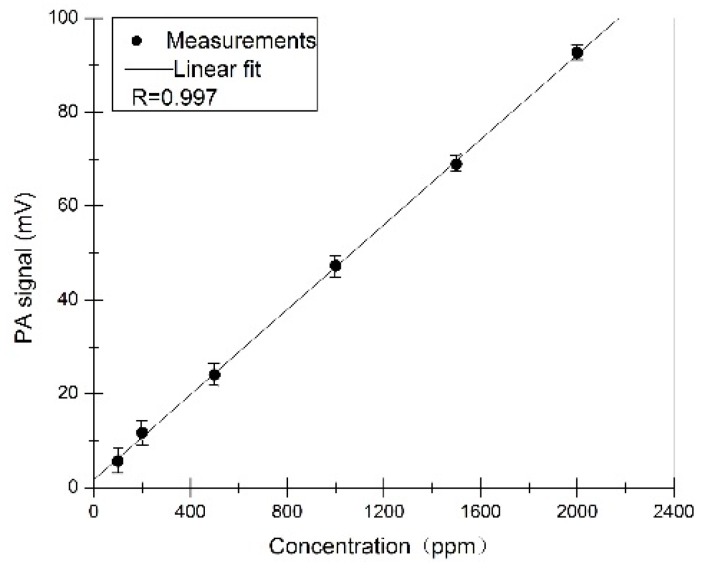
PA signals to different concentrations. Error bars give the range of the corresponding PA signals.

To test the sensor performance, different concentrations ranging from 100 ppm to 2000 ppm were prepared by diluting certified methane with the gas sample preparation unit. The PA signal amplitude of different concentrations was measured as seen in Figure 8. Each dot is an average of 10 individual measurements. A linear fit was applied to obtain the linearity of the sensor. Experiments show that the PA sensor has good performance.

**Figure 9:**
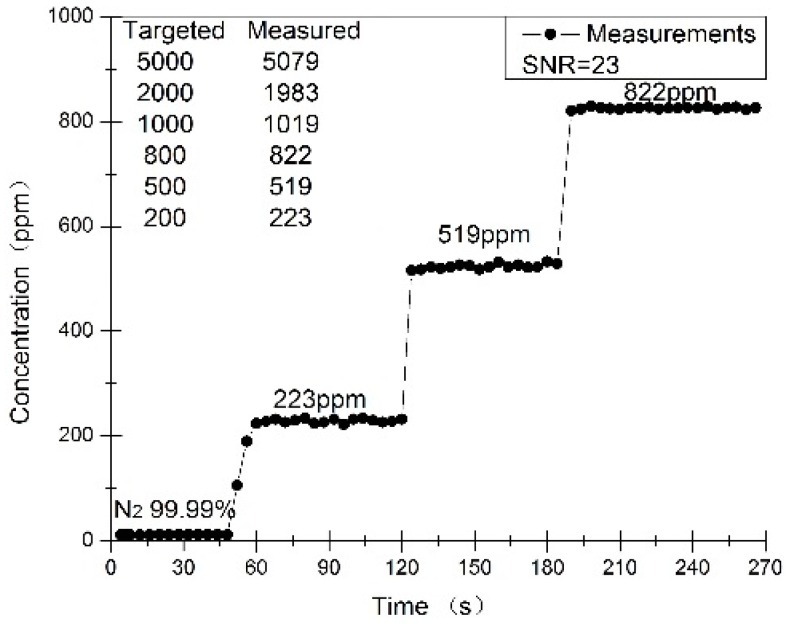
Measured dilution process of the 5000 ppm CH_4_ reference concentration.

The detection limit and response time of this PA methane sensor were evaluated by detecting the PA signal with diluting calibrated mixture of 5000 ppm CH_4_ in air. Both targeted and detected CH_4_ concentrations were demonstrated as shown in Figure 9. The standard deviation (σ) of the PA signal is 0.52. The PA signal amplitude of 223 ppm is 11.9 mV, so a SNR of 23 was calculated, which indicated that the detection limit (SNR = 1) of this PA sensor for CH_4_ is about 10 ppm. The response time is about 4 s. There are some differences between the targeted gas and measured CH_4_ concentrations. One of the main reasons is most likely the precision of the gas sample preparation unit during the dilution process.

**Figure 10:**
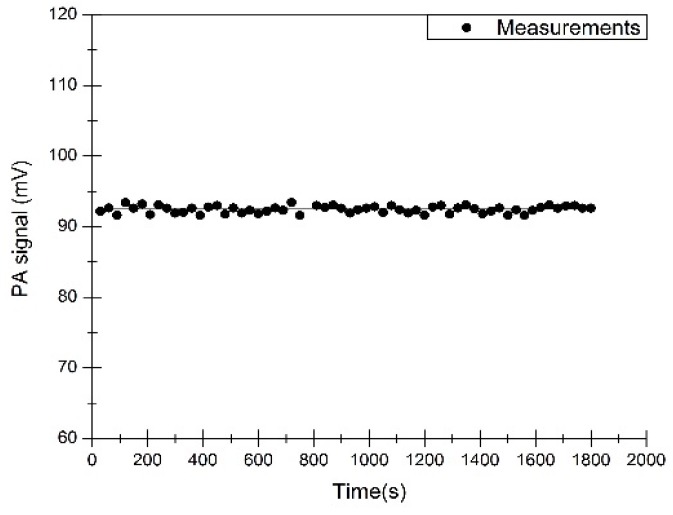
Continuous measurements of 2000 ppm CH_4_.

The PA signals of 2000 ppm CH_4_ were measured for evaluating measurement stability of this PA sensor (see Figure 10). The CH_4_ concentrations were displayed in digital display, for which these values were recorded at intervals of 30 s. Its relative error is less than 0.5%. The measurement results thus show that this sensor has good stability.

## 4. Conclusions

A new gas sensor was developed, which uses a DFB diode laser, FPGA lock-in, and resonant photoacoustic spectroscopy. The proposed PA methane sensor is portable, compact, easy operation and intuitive display. A minimum detection limit of 10 ppm for CH_4_ was demonstrated at atmospheric pressure. Further improvements are carried out for improving the detection sensitivity by optimizing the design for the FPGA lock-in. This sensor shows the possibility of providing a new instrument for coalmine methane detection, environmental monitoring and industrial applications in combination with appropriate detection schemes.
